# E. Henry Neall, D.D.S.

**Published:** 1900-09

**Authors:** 


### Obituary.

### E. HENRY NEALL, D.D.S.

On Sunday morning, July 8, there died one of Philadelphia's well-known dentists, Dr. E. Henry Neall, in the sixty-third year of his age, at his late residence, 114 East Washington Lane, Germantown.

Dr. Neall studied the art of dentistry in the office of his father, Dr. Elijah M. Neall, a pioneer in the profession. His specialty was the carving and baking of block teeth, and he was recognized as an expert along that line. As late as 1899 he gave demonstrations in that difficult dental branch before the students of the Medico-Chirurgical College.

Practising long before a college diploma was thought necessary, Dr. Neall, nevertheless, recognizing the advantage of possessing the same, matriculated in the Pennsylvania College of Dental Surgery, from which he graduated in 1868.

Many little devices and labor-saving tools, in connection with his beloved profession, can be traced to his ingenuity and fertile brain. He was ever active in dental society work, and was a member of the old Pennsylvania Society, the Odontological Society, and the Pennsylvania State Dental Society. Besides this, he gave of his services at frequent intervals to the dental students, being upon the clinical staff of both the University of Pennsylvania and the Medico-Chirurgical College.

Dr. Neall was a Christian gentleman in the fullest sense of the word. He was a member of Calvary P. E. Church, Germantown, and an earnest worker in the Brotherhood of St. Andrew.

During the war he went to the front with the Christian Commission to relieve the sick and suffering soldiers.

Dr. Neall was twice married, his first wife being Miss Elizabeth Enyard Montgomery, of Philadelphia; his second wife, formerly Miss Emily L. White, also of Philadelphia, survives him.

He leaves six children by his first wife,—Dr. Walter H. Neall, Mrs. W. K. Matsinger, Robert M. Neall, Mrs. Charles J. Pilling, Benjamin T. Neall, and Miss Edith Neall.

